# MetaSeeker: sketching an open invisible space with self-play reinforcement learning

**DOI:** 10.1038/s41377-025-01876-0

**Published:** 2025-06-04

**Authors:** Bei Wu, Chao Qian, Zhedong Wang, Pujing Lin, Erping Li, Hongsheng Chen

**Affiliations:** 1 ZJU-UIUC Institute, Interdisciplinary Center for Quantum Information, State Key Laboratory of Extreme Photonics and Instrumentation, Zhejiang University, Hangzhou, China; 2 ZJU-Hangzhou Global Science and Technology Innovation Center, Zhejiang Key Laboratory of Intelligent Electromagnetic Control and Advanced Electronic Integration, Hangzhou, China; 3 Jinhua Institute of Zhejiang University, Zhejiang University, Jinhua, China; 4 Zhejiang Province Key Laboratory of Intelligent Vehicle Electronics, Hangzhou Dianzi University, Hangzhou, China

**Keywords:** Metamaterials, Other photonics

## Abstract

Controlling electromagnetic (EM) waves at will is fundamentally important for diverse applications, ranging from optical microcavities, super-resolution imaging, to quantum information processing. Decades ago, the forays into metamaterials and transformation optics have ignited unprecedented interest to create an invisibility cloak—a closed space with any object inside invisible. However, all features of the scattering waves become stochastic and uncontrollable when EM waves interact with an open and disordered environment, making an open invisible space almost impossible. Counterintuitively, here we for the first time present *an open, cluttered, and dynamic* but invisible space, wherein any freely-moving object maintains invisible. To adapt to the disordered environment, we randomly organize a swarm of reconfigurable metasurfaces, and master them by MetaSeeker, a population-based reinforcement learning (RL). MetaSeeker constructs a narcissistic internal world to mirror the stochastic physical world, capable of autonomous preferment, evolution, and adaptation. In the perception-decision-execution experiment, multiple RL agents automatically interact with the ever-changing environments and integrate a post-hoc explainability to visualize the decision-making process. The hidden objects, such as vehicle cluster and experimenter, can freely scale, race, and track in the invisible space, with the environmental similarity of 99.5%. Our results constitute a monumental stride to reshape the evolutionary landscape of metasurfaces from individual to swarm intelligence and usher in the remote management of entire EM space.

## Abstract (web summary)

MetaSeeker controls a swarm of metasurfaces for the creation of an open electromagnetic invisible space.

## Graphical abstract

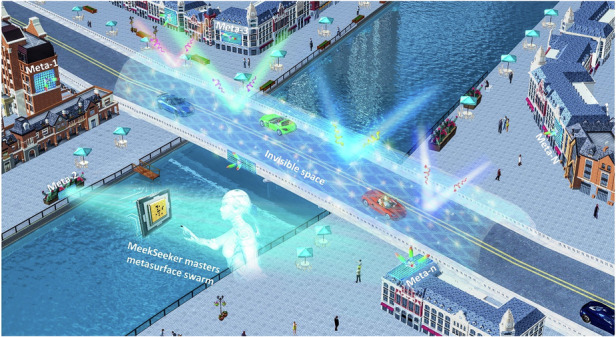

## Introduction

Whether it is the radio shielding of spacecraft instruments, the natural evasion of predator attacks or the network immunity to noise interferences, in all of these cases freely controlling EM waves is highly demanded to render an object invisible. A direct physical picture is that the external detectors or naked eyes cannot perceive the existence and movement of the hidden objects. This dream was considered almost impossible until the advent of metamaterials^1,2^ and their planar equivalences, metasurfaces^3,4^ two decades ago. As a class of artificially engineered materials, they bring a new twist to craft EM waves at subwavelength scale by flexibly designing the geometric configurations^5^, material compositions^6^, and spatiotemporal distributions^7^. From the theoretical perspective, many cloaking methods have been successively proposed, such as near-zero refractive index^8^, scattering cancellation^9^, and topological protection^10^. Of particular interest is the transformation optics^11^ that can perfectly guide the flow of light by cladding an anisotropic and inhomogeneous metamaterial shell. Notably, recent advancements in reconfigurable/active and intelligent metasurfaces have further bestowed invisibility cloak a tunable range to cater for a number of agile applications^12–17^.

A common feature for all previous works is to clad an interested object with a carefully-designed metamaterial coating or metasurface inclusion to formulate a closed space, greatly hindering its flexibility and maneuverability. Moreover, the tremendous difficulties in practical realization force scientists to make various parameter approximations and model simplifications, restricting the experiment to a predefined object and to a static, pure, and idealistic scene^18–20^. Here, we start from a completely different view, that is, is it possible to directly render an open and cluttered space invisible? It ideally requires that any quantity, geometrical size, external shape, material composition, and movement trajectory of the interested objects inside such space cannot produce any nuanced EM disturbances exposed to outside detectors. With this superpower, it would be very exciting to remotely empower arbitrary space and modify the EM environment. However, there are many challenges to achieve this goal. First, complex EM scattering in inenarrable, disordered, and dynamic environments is unlikely to be described with concrete mathematical formulas and physical models. It reveals the main challenge in multi-agent coordination that requires high-level algorithm modelling to control a group of metasurfaces to quickly adapt to dynamic environments. Second, in stark contrast to passive and tunable cloaking in which the interested objects are predefined, the agnostic and non-stationary objects bring many uncertainties to the invisible space, necessitating meticulous metasurface design, systematic deployment, and context awareness.

Here we introduce a team of metasurfaces that collectively creates an EM invisible but open space in a highly cluttered environment^21,22^. Multiple reconfigurable metasurfaces can be noninvasively deployed and freely distributed in the surrounding settings, either inside or outside^23^ the invisible space. Traditional inverse design of metasurfaces typically rely on deep learning, which requires manually created datasets. However, deep learning is suited for interpolation rather than extrapolation, meaning it often fails to generalize beyond the scope of the dataset. This limitation becomes particularly problematic when dealing with optimization problems that involve a high degree of freedom, as the dataset may not encompass all possible variations, leading to incomplete or biased knowledge. To efficiently master the distributed metasurfaces, we propose MetaSeeker, short for seeking in the metasurface space with a population-based RL model. MetaSeeker utilizes a population of RL agents that construct an internal world to mirror the physical world in a unique way, facilitating a comprehensive grasp of intricate physical dynamics in the disordered systems within an exceptionally brief timeframe. Equipped with a comprehensive perception-decision-execution accessory, multiple agents autonomously interact with the physical world, conducting on-site learning based on far-field feedback. Such self-optimization mechanism enables robust handling of extremely rich inputs in metasurface space and kaleidoscopic environments. Repeated evaluations suggest that the detected far-field signals maintain stable when different hidden objects freely move inside the EM invisible space. To explore the internal world, MetaSeeker integrates a post-hoc explainability to elucidate its decision-making process, making a big step toward “white-box” artificial general intelligence (AGI). The advent of MetaSeeker may redefine our understanding of once-fictional concepts, transcending imagination to manifest tangible realities, such as the manipulation of warp engine time and the harnessing of black hole energy.

## Results

### Teaming up distributed metasurfaces in a disordered environment

**Fig. 1:**
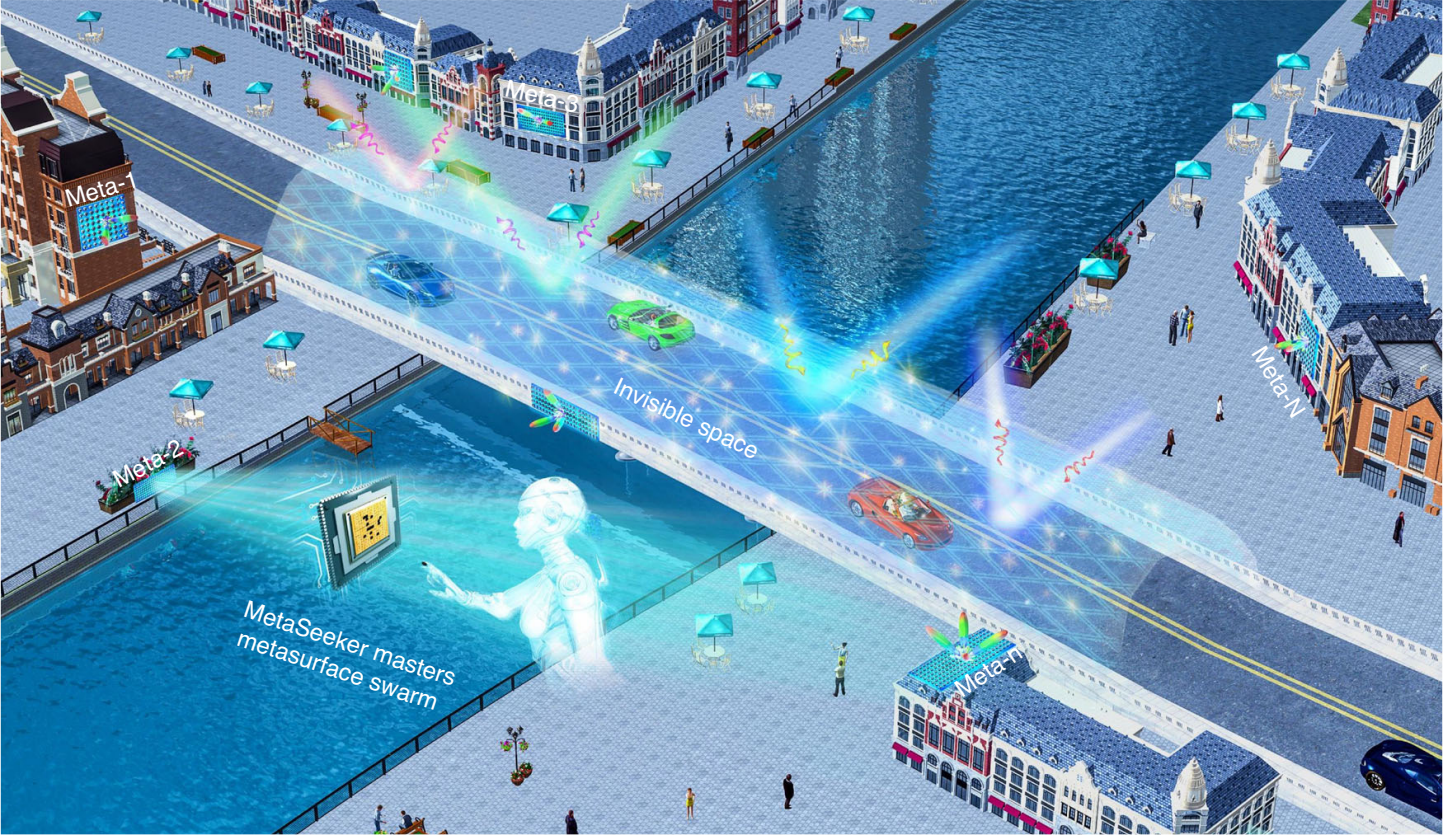
Metasurface swarm for the creation of an EM invisible space. A team of metasurfaces is randomly distributed amidst urban landscapes and decorated on the surfaces of buildings, bridges, and other infrastructures. They work synergistically to create an EM invisible space, which means any moving vehicles, pedestrians, animals, and other entities in such space will be rendered imperceptible simultaneously to external detections, as if there were purely stationary background. Metasurface swarm is mastered by MetaSeeker, a population-based RL model that constructs an internal world to mirror the physical world in its unique way, facilitating a comprehensive grasp of intricate physical dynamics in disordered systems within an exceptionally brief timeframe

Figure 1 depicts a surrealistic vision of metasurface swarm, which is randomly distributed amidst urban landscapes and decorated on the surfaces of buildings, bridges, and other infrastructures. Each metasurface comprises a great number of reconfigurable meta-atoms to independently adjust its local reflection coefficient for a user-defined goal at the physical level. Controlling EM wave in this disordered environment begets contemplation of numerous practical applications, such as wireless communication^24,25^, non-line-of-sight imaging^26^, probabilistic detection and ranging^27^. Here we set the goal as the creation of an EM invisible space, as exemplified in the covered tunnel in Fig. 1; it means that any moving vehicles, pedestrians, animals, and other entities in such space will be rendered invisible simultaneously to external detections, as if there were stationary background. We want to emphasize that this is different from and far beyond conventional metamaterials-based invisibility cloaks because they work only for predefined, stationary objects, and the metamaterial coating must be tightly clad with hidden object^12^. Teaming up multiple metasurfaces has the potential to couple respective advantages and adapt to agile applications, thus getting rid of the constraints posed by conventional stand-alone metasurface working modality. Enabling swarm vision in electromagnetism requires parallel developments of a number of key technologies, amalgamating strong artificial intelligence (AI), system automatic control, EM scattering theory, and reconfigurable metasurfaces. To efficiently master the distributed metasurfaces, we devise MetaSeeker, aligning multiple RL agents to optimize the EM invisible space. Even in the case of damage or malfunction of metasurfaces, the remained metasurfaces can autonomously recalibrate to redress deficiency and recover original functionality.

### Mastering distributed metasurfaces with MetaSeeker

The optimization of a team of metasurfaces presents a significant challenge due to the extremely high design degrees of freedom. For example, in this work, the optimization complexity reaches 10^96^, even surpassing the total number of atoms in the universe. Existing inverse design methods pin their hopes on simplified and low-dimensional objects, which fail to be applied in distributed metasurfaces. We endeavor to develop AGI to optimize metasurfaces for near-perfect invisibility performance. The term “AGI” denotes an AI system capable of emulating human-level intelligence, proficient across various domains rather than being specialized in a singular field^28^.

The emergence of the MuZero algorithm^29^ in 2020 marked a stride toward AGI. MuZero, a model-based RL, achieved champion-level proficiency in 57 Atari games^30^ and demonstrated superhuman performance in Go, chess, and shogi. However, its limitations lie in handling visually complex inputs, navigating continuous action domains, and lacking interaction with the physical world, which restrict its practical applications to gaming scenarios. Building upon MuZero’s foundation, our innovation advances a comprehensive and cross-domain strategy.

**Fig. 2:**
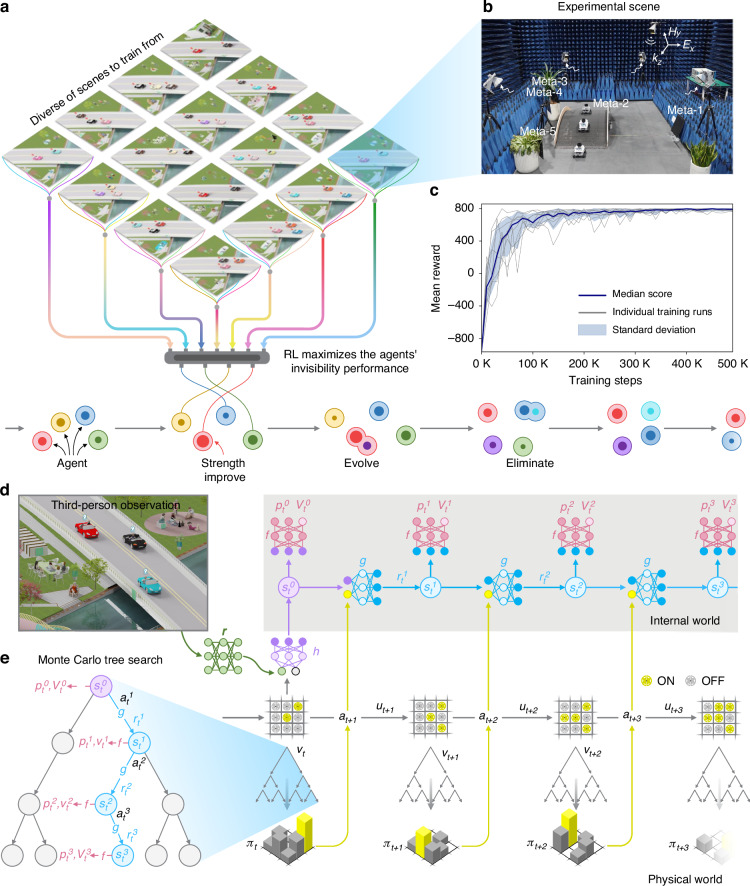
MetaSeeker architecture. **a** The two-tier optimization strategy. Agents generate the training data by engaging in metasurface-board games across a variety of scenes. Leveraging the training data, a group of independent RL agents are concurrently trained to optimize the metasurfaces. Additionally, PBT is utilized to optimize the RL procedure itself. As depicted in the lower section, each circle symbolizes an agent in the population, where the inner circle’s size reflects its strength. Agents evolve (represented as splitting) with descendants inheriting and mutating hyperparameters (represented as color). **b** An example of experimental scene in a microwave anechoic chamber. To manipulate the EM waves, a swarm comprising five strategically concealed metasurfaces works synergistically. **c** The mean reward of agents in the PBT process. The dark line signifies the median score across 10 agents, light lines indicate individual agents, and the shaded region represents the standard deviation across the agents. **d** The processing of an agent for a temporal sequence of invisible scene images and optimization for the distributed metasurfaces. The model of MetaSeeker integrates four interconnected components for recognition (*r*), representation (*h*), dynamics (*g*), and prediction (*f*). The model is recurrently unrolled for *K* steps and iteratively executes these components until the end of the episode, then stores the trajectory data in a reply buffer. The parameters of the recognition, representation, dynamics and prediction networks are jointly trained end-to-end by backpropagation through time to predict three crucial quantities: the policy ${p}_{t}^{k}$ ≈ *π*_*t*+*k*_, value ${v}_{t}^{k}$ *≈ z*_*t*+*k*_ and reward ${r}_{t}^{k}$ *≈ u*_*t*+*k*_. **e** The schematic diagram of MCTS, a guiding mechanism for agent’s actions

Specifically, we adopt a two-tier optimization approach by incorporating model-based RL and population-based training^31^ (PBT), as depicted in Fig. 2a. PBT adjusts the hyperparameters of RL procedure (Dirichlet exploration noise, regularization parameter, and learning rate) by replacing underperforming agents with mutated versions of superior agents. This autonomous process enables the continual improvement, evolution, and adaptation of agents within the population, aligning seamlessly with Darwin’s theory of evolution and eliminating the need for human intervention.

In our task, MetaSeeker aims to optimize the distributed metasurfaces, with each meta-atom toggling between ON and OFF states, as depicted in Supplementary Fig. 1. Inspired by the related model’s proficiency in board games^29,32^, the metasurface optimization is liken to board gameplay. This involves adjusting meta-atom’s phase based on piece placement, aiming to maximize invisibility performance.

The model of MetaSeeker *μ*_*ϑ*_ with parameters *ϑ* is represented by a combination of a recognition network *r*_*ϑ*_, a representation network *h*_*ϑ*_, a dynamics network *g*_*ϑ*_ and a prediction network *f*_*ϑ*_, as depicted in Fig. 2d. Operating at each time *t* across *k* = 0, …, *K* steps, the recognition network combines a pretrained (frozen) Mask-RCNN^33^, whose goal is to perform semantic segmentation on third-person observations, with an encoder, enriching agents’ comprehension of the experimental environment and facilitating the processing of visually rich inputs. The representation network *h*_*ϑ*_ takes past observations (*o*_*1*_, …, *o*_*t*_) as input, comprising encoded recognition images and metasurface-boards, and transforms them into the “root” internal state ${s}_{t}^{0}$, i.e., ${s}_{t}^{0}$ = *h*_*ϑ*_(*o*_*1*_, …, *o*_*t*_). It serves as a channel to mirror the real physical world to the virtual internal world of agents. In this internal world, the agents reason abstractly and think creatively like humans do, just like creating a human-like brain in a machine. The dynamics network *g*_*ϑ*_ then iteratively updates the internal state ${s}_{t}^{k}$ and computes an immediate reward ${r}_{t}^{k}$ through a recurrent process that takes the previous internal state ${s}_{t}^{k-1}$ and a hypothetical next action ${a}_{t}^{k}$ as input, i.e., ${r}_{t}^{k}$, ${s}_{t}^{k}$ = *g*_*ϑ*_(${s}_{t}^{k-1}$, ${a}_{t}^{k}$). At each step, the prediction network *f*_*ϑ*_ produces a policy ${p}_{t}^{k}$ (adjusting a meta-atom) and a value ${v}_{t}^{k}$ (predicting the final invisibility performance in this episode), i.e., ${p}_{t}^{k}$, ${v}_{t}^{k}$ = *f*_*ϑ*_(${s}_{t}^{k}$). Exact network architecture is described in Supplementary Fig. 2. Unlike traditional approaches to model-based RL^34^, the internal states are free to represent any state that accurately estimates the policy, value, and reward, without attaching any semantics of the environment state. This characteristic significantly reduces the information that the model needs to maintain, and improves the adaptability across diverse domains of expertise.

Having established such an interconnected network, we employ Monte Carlo tree search^35^ (MCTS) to explore hypothetical future trajectories ${a}_{t}^{1}$, *…*, ${a}_{t}^{k}$ given past observations *o*_*1*_, *…*, *o*_*t*_, as shown in Fig. 2e. At each internal node, the search tree combines the policy, value and immediate reward predicted by the current model *μ*_*ϑ*_ using lookahead search^36^, producing an improved policy *π*_*t*_ and value *v*_*t*_ at the root of the search tree after 200 simulations. The next action *a*_*t+1*_ ≈ *π*_*t*_ is then chosen by the search policy and serves as the input for the dynamics network at the next step.

We employ PBT to concurrently train a group of independent agents, enabling swift adaptation across diverse scenarios via a self-optimization mechanism. Figure 2c illustrates the mean reward of agents over 500,000 training steps. The results elucidate a discernible enhancement in the invisibility performance of each agent as training processes, culminating in a state of saturation.

### Perception-decision-execution experimental system

**Fig. 3:**
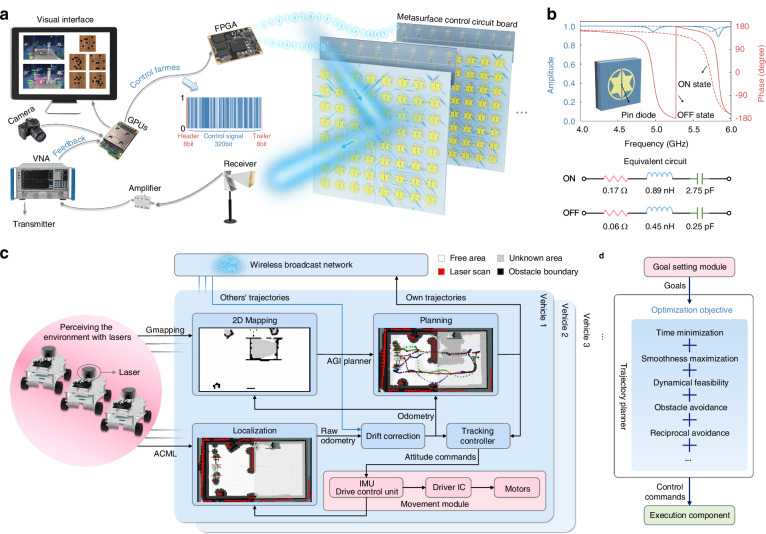
Perception-decision-execution experimental setup and metasurface design. **a** Schematic of agents interacting with physical world. The pivotal experimental components include a camera for perception, GPUs for decision-making, metasurfaces for action, and receiving antennas for feedback. They collaborate to create a 3D EM invisible space in real world. Specifically, agents translate the observations from virtual metasurface-boards into the control frames for metasurface entities, and then transmit them to FPGA via serial communication. These control frames comprise three segments, i.e., header (8 bits), control signal (320 bits) and trailer (8 bits). Subsequently, the FPGA dispatches these control signals to the serial-to-parallel circuit boards connected behind each metasurface panel, thereby autonomously adjusting each meta-atom. **b** The dispersion relation of the reconfigurable metasurfaces. A PIN diode is welded inside the meta-atom to enable tunable reflection coefficient; see its effective “R-L-C” model. The detailed geometries are left in Supplementary Fig. 5. **c** The system architecture of vehicular cluster. Each vehicle is equipped with perception, localization, planning, and control functionalities. They are loosely coupled through a wireless broadcast network to share trajectories. **d** The framework of trajectory planner. It aims to navigate vehicles to any designated positions, adhering to essential constraints such as time minimization, smoothness maximization, dynamical feasibility, obstacle avoidance, and reciprocal avoidance

We randomly design the disordered experimental environment in a microwave anechoic chamber (Fig. 2b) and develop a comprehensive suite of intelligent systems that integrate perception, decision, action, and feedback modules, as illustrated in Fig. 3a. These components collectively empower the intelligence algorithm to interact with the EM world autonomously without any human intervention or prior dataset preparation.

For the perception component, we place a fixed viewpoint camera to capture third-person observations of the experimental scenes. These acquired images undergo semantic segmentation to discern the categories, positions and contours of interferences, as depicted in Supplementary Fig. 3. For the decision and action components, MetaSeeker is proposed to optimize the reflection state distributions of metasurfaces. This algorithm is run on Graphics Processing Units (GPUs). MetaSeeker calculates voltage control signals for metasurfaces and transmits them to a field programmable gate array (FPGA) via serial communication. Subsequently, the FPGA dispatches these signals to serial-to-parallel circuit boards connected behind each metasurface, thereby autonomously manipulating individual meta-atoms. The reflection coefficient of the meta-atom is shown in Fig. 3b, with solid lines representing OFF state (characterized by inside PIN diode cutoff) and dashed lines denoting ON state (characterized by inside PIN diode conduction). The optimization process of metasurfaces is analogized to playing board games. Each metasurface has two phase states at the operating frequency (5 GHz), i.e., 0 and π. Initially, the phases of metasurfaces are all-zero. The voltages applied to the meta-atoms, where agents place pieces, are then adjusted to 3.3 V to induce a phase shift to π. For the feedback component, agents adjust policies based on the far-field intensities detected by receiving antennas, dynamically adapting to shifting interferences and disordered environments. To increase the signal-to-noise ratio of the measured intensities, we installed a low-noise amplifier with a gain of 23 dB between the vector network analyzer and the receiving horn antenna. This confirms the reliability of the measured far-field intensities, demonstrating minor disturbance from background noise.

In order to mimic real-world urban scenarios and circumvent the labor-intensive process of artificially diversifying the environment, we have engineered a fully autonomous vehicular cluster. Moreover, to further validate the algorithm’s generality, we continue to use MetaSeeker for trajectory planning of each vehicle. Coincidentally but reasonably, our designed vehicles share a resemblance to bats capable of navigating freely in the dark while avoiding obstacles and other moving entities. A notable consistence lies in the use of radar technology for perception; much like bats employing biological sonar by emitting ultrasonic waves to sense their surroundings, our vehicles utilize radar sensors to perceive the environment. Moreover, bats exhibit a remarkable ability to swiftly plan the most effective path in complex environments to avoid obstacles and capture prey, thus we propose a trajectory planner for multi-objective optimization. Beyond the capability of bats, we take a step further by loosely coupling all vehicles through a wireless broadcast network to share information^37^. Our method embraces both individual and cluster intelligence. As Murphy^38^ highlighted, weakly centralized, distributed organization of the cluster exhibits heightened robustness and resilience, capable of retaining functionality even in scenarios where communication and global positioning system (GPS) signals are lost.

### Experimental demonstration: scalability, racing, and tracking

Our experiments conducted in a microwave anechoic chamber affirm Murphy’s concept, where GPS signals are obstructed. The vehicular cluster, deprived of GPS signals, relies exclusively on laser scans for spatial awareness, and communicates through a wireless broadcast network. Three proof-of-concept experiments authenticate the invisible space: scalability, racing, and tracking (Supplementary Movie S1). In these experiments, the working frequency is 5 GHz, and the far-field intensities measured in the static reference scenes serve as targets, with values at four detection angles being −57.5 dB, −65.3 dB, −64 dB, and −66.3 dB, respectively. These scenes include a bridge, plants, and necessary detection devices.

**Fig. 4:**
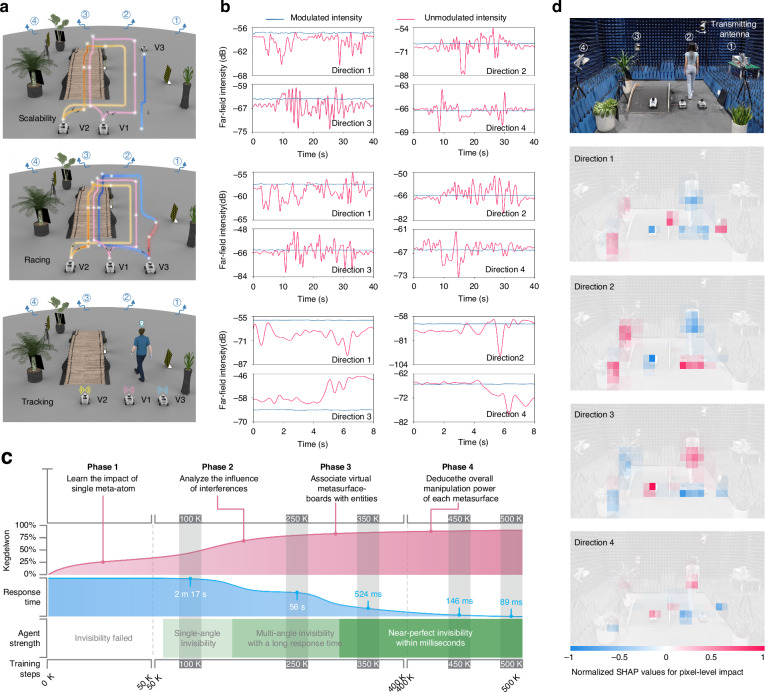
Dynamic experiment demonstration and interpretability analysis. **a** Visualization of the vehicular cluster’s trajectories across three different tasks, i.e., scalability, racing and tracking. The darkness of the trajectory color for the slave vehicles (V2 and V3) signifies their distance behind the leader vehicle (V1)—darker color indicates a greater distance, while lighter color indicates closer proximity. The red line highlights the trajectory segment where the slave vehicle overtakes the leader vehicle. **b** Far-field measurement along four distinct directions with incident frequency of 5 GHz. The blue/red lines represent the far-field intensities measured with/without MetaSeeker driven metasurfaces. The results confirm that intelligent distributed metasurfaces effectively ensure the constancy of far-field intensities at multiple detection angles, unaffected by moving objects. **c** Progression of MetaSeeker during training. Depicted is the progression of knowledge representation and invisibility performance of MetaSeeker over 500,000 training steps, segmented into four distinct phases. **d** Interpretability analysis of MetaSeeker. The topmost image displays the original third-person observation at a specific time step, and the subsequent images delineate the pixel-level impact on far-field intensities at four detection angles. We analyze the interpretability using SHAP values, where red/blue pixels signify regions where the objects increase/decrease the far-field intensity

Initially, we validate the cluster scalability to ensure the uninterrupted invisibility performance even if a vehicle enters the invisible channel halfway. At the beginning, only two autonomous vehicles are in motion, and at t = 21 s, the third vehicle positioned near the EM absorbing materials joins the team, forming a triangular formation as the three vehicles move synchronously. The top graph of Fig. 4b illustrates the temporal variation of far-field intensities during vehicular motion, demonstrating that the distributed metasurfaces maintain robust immune performance across various detection angles. For an illustrative purpose, the mean absolute percentage error (MAPE) is employed as a metric to quantify the immune performance, revealing that the invisibility performance at four detection angles reaches up to 99.73%, 99.74%, 99.77%, and 99.95%, respectively. Results attest the formidable anti-interference capabilities of the invisible space, impervious to varying interference levels.

Moving on to autonomous racing, a common scenario on real-world roadways, the far-field intensities, depicted in the middle of Fig. 4b, exhibit distinct patterns. Without the metasurface modulation, significant far-field fluctuation occurs due to moving interferences. In contrast, with the metasurface modulation, the far-field intensities maintain stable, emulating an EM environment devoid of interferences. Specifically, the invisibility performance at four detection angles is 99.93%, 99.76%, 99.87%, and 99.8%, respectively. Subsequently, we conduct cluster tracking experiment to validate the robustness of the EM invisible space against various interference modalities. The results are depicted at the bottom of Fig. 4b, revealing that the invisibility performance at four detection angles reaches up to 99.82%, 99.52%, 99.78%, and 99.89%, respectively. Results demonstrate that, even in the presence of human interference, the distributed metasurfaces adeptly collaborate to shape EM beams, neutralizing the impact of diverse interferences on far-field intensities.

**Fig. 5:**
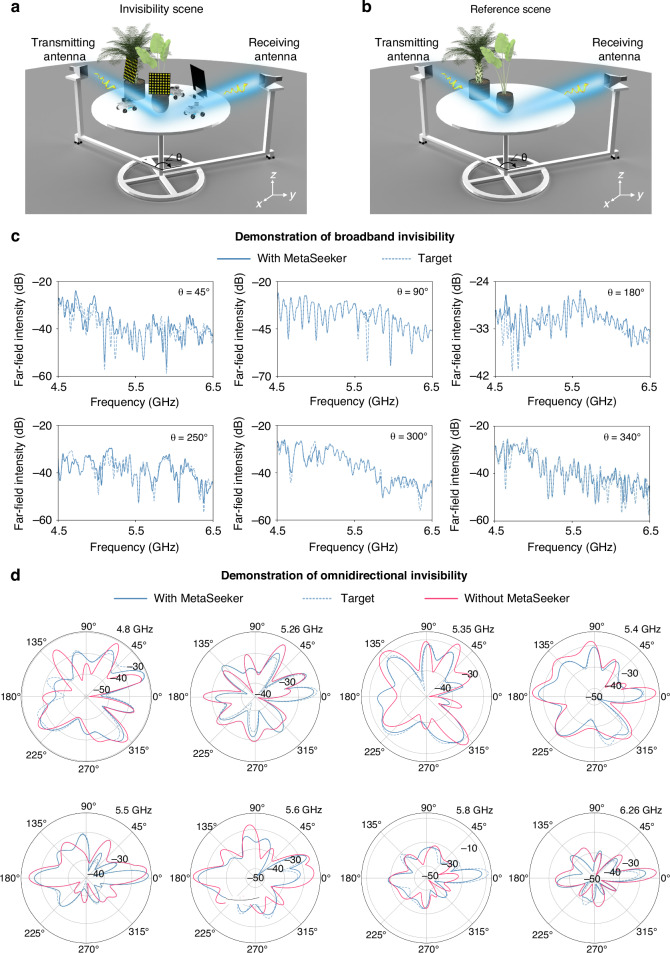
Experimental demonstration of broadband and omnidirectional invisibility. **a** The experiment setup on a turntable. By controlling metasurfaces, all vehicles and metasurfaces can be rendered invisible, making the detected broadband far-field intensities similar to pure background. **b** The reference scene. **c** Demonstration of broadband invisibility at various detection angles. The solid line represents the far-field intensities optimized by MetaSeeker in the invisibility scene, and the dashed line shows the target far-field intensities from the reference scene. **d** Demonstration of omnidirectional invisibility at different incident frequencies. The blue/pink solid line represents the far-field intensities with/without MetaSeeker, and the blue dashed line represents the target

To further demonstrate the invisibility over a certain bandwidth and arbitrary detection angles, we conducted experiments on a turntable setup. Two plants, three vehicles, and three metasurfaces were placed on a circular platform, with the transmitting and receiving antennas mounted on rotatable bracket to simulate random detection scenario (Fig. 5a). The optimization goal is to ensure that the detected broadband far-field intensities match those of the reference scene (Fig. 5b), regardless of the receiving antenna’s rotation angle. By rotating the bracket, we changed the angle *ϑ* between the antennas and compared the far-field intensities in the two scenes over the frequency range from 4.5 GHz to 6.5 GHz. Figure 5c shows the broadband invisibility at different detection angles. It turns out that MetaSeeker achieved an average invisibility bandwidth of over 1.5 GHz at various detection angles. This bandwidth is contributed by the global effect of all distributed metasurfaces, each of which has slightly different working frequency. Figure 5d illustrates omnidirectional invisibility at different incident frequencies, showing that the distributed metasurfaces perform well at any given angle. More experimental results are left in Supplementary Fig. 4. These experiments demonstrate that a swarm of narrowband metasurfaces can effectively achieve broadband invisibility. Although the metasurfaces exhibit strongest modulation capability at the resonant frequency, they can still modulate EM waves, albeit to a lesser extent, even when the operational frequency deviates from resonance. Furthermore, the cooperative interaction among multiple metasurfaces enhances global control over the EM waves, facilitating modulation across a broader frequency range.

Further improvements of MetaSeeker could include deploying more metasurfaces to enhance the degree of freedom, and replacing the metasurfaces with broadband structures, thereby expanding the bandwidth and detection angle range. In addition, we can deploy an intelligent EM detector^39^ to perceive incident information related to frequency, polarization, and angle in real-time. The predicted data can then serve as input for MetaSeeker to integrate the incident wave knowledge, enabling quick adjustments to metasurfaces in response to rapidly changing detections.

### Interpretability analysis using Shapley additive explanations

We hypothesize that the agents achieving such exceptional proficiency have assimilated a nuanced comprehension of EM invisibility. Shapley additive explanations^40^ (SHAP), a game-theory-inspired method, can enhance interpretability by computing importance values for prediction features. By employing SHAP values, we analyze MetaSeeker’s mastery of diverse facets of invisibility knowledge—embracing superficial insights, such as the impact of metasurface’s phase alteration, interference’s occurrence on far-field intensities, as well as profound understandings like associating virtual metasurface-boards with entities in the third-person observations and deducing the overall manipulation power of each metasurface on EM waves.

Figure 4c illustrates MetaSeeker knowledge representation and response time development over 500,000 training steps; see Supplementary Movie S2. As training progresses, MetaSeeker ascends from a grasp of superficial insights to profound understanding, paralleled by an augmentation in immune/invisibility performance and a reduction in response time. Ultimately, it achieves near-perfect immune effect within milliseconds, allowing the decision-making process to be disregarded. We conduct an interpretability analysis on MetaSeeker, as depicted in Fig. 4d, with red and blue pixels signifying regions where the objects respectively increase and decrease the far-field intensity. The analysis elucidates that the antagonistic influences of interferences and metasurfaces are capable of counterbalancing each other (intricate scattering cancellation^9^) on the far-field intensities.

In the interaction between AI and electromagnetism, the significance of discerning the rationale behind model predictions rivals the accuracy of the predictions themselves. This endeavor serves to illuminate the intricacies of physical dynamics, enhance the transparency and credibility of decision-making, and unveil novel physical laws. Nevertheless, a discernible trade-off exists between a model’s performance and its interpretability. Specifically, the escalating complexity of hierarchical structure amplifies predictive capabilities but renders explanations elusive, hence earning the moniker “black-box” model. Attempts to demystify this black-box, such as incorporating fundamental physical laws, have hitherto proven futile. Inherent limitations stem from their working mechanisms, such as insufficient physical theories for highly cluttered media or overly intricate physical processes obfuscating model comprehension. Our proposed post-hoc explainability method circumvents direct interference with the model’s training process, focusing instead on interpreting its decision-making post-training. It seamlessly integrates with models boasting arbitrary hierarchical complexity and presents significant utility for immutable pre-trained models. This innovative method facilitates an intuitively visualized invisible space, pioneering the implementation of a profoundly intricate “white-box” model within the realm of physics, and providing opportunities for comprehending and governing strong AI.

## Discussion

Our results demonstrate a team of metasurfaces that orchestrates the free management of EM waves through intricate internal scattering^41–44^, effectively concealing any moving objects in the designated 3D space. It brings available metasurfaces from individual to swarm intelligence and overcome the constraints in conventional cloaking techniques confined by stationary environment, individual modality, and lack environmental compatibility. In practice, the working environment is very complicated, unstable, and occluded, making it impossible to be characterized with a closed-loop and even semi-known analytical model. In this regard, swarm intelligence becomes extremely urgent, shifting the attention from idealistic physical toolsets to human-level intelligent system.

MetaSeeker, a population-based RL model, constructs an internal world to mirror the physical world in its unique way. In the internal world, it engages in abstract reasoning and creative contemplation, embodying a machine-rendered emulation of the human brain but with vastly superior intelligence. Through the PBT strategy, a group of RL agents autonomously improve, evolve, and adapt by replacing underperforming agents with mutated superior versions, adhering to Darwin’s evolutionary principles for swift cross-domain adaptation. Equipped with the perception-decision-execution “limbs”, agents autonomously interact with the environment, engaging in on-site learning facilitated by real-world feedback within remarkably condensed time frames.

Human endeavors are deliberately eschewed in both the data preparation and learning phase of MetaSeeker, in accordance with our conviction that transcending human intellect necessitates a departure from homologous thought paradigms. The results robustly validate our proposition, which showcase the model’s exceptional proficiency in domains such as gaming^29^, reasoning^45^, electromagnetism^46–51^, and robotics, fostering unwavering confidence in its unparalleled capability for real-world AI applications. When deploying MetaSeeker in real-world environments, its invisibility performance is primarily limited by the metasurface swarm’s ability to modulate EM waves, requiring careful design to balance dynamic interference compensation. Additionally, MetaSeeker utilizes image recognition to identify interference, and expanding the image dataset with diverse real-world objects is recommended to improve robustness and applicability. Significance of our work not only lies in the field of invisibility cloak, but also spurs a myriad of hitherto inaccessible physical concepts to practical applications, including inter-satellite communication and detection in disordered systems.

## Materials and methods

### Experimental setup details

A fixed viewpoint camera (HIKVISION DS-UVC-U178R) is positioned to capture the third-person observations of the experimental environment. The images captured by this camera are transmitted in real-time to a computer via a USB connection cable. he computer processes the input data and generates voltage control signals for the metasurfaces, which are then transmitted to a field-programmable gate array (FPGA) via serial communication. Specifically, communication between the computer and FPGA is facilitated through a USB-to-serial converter (CH340). The protocol uses a header byte (0xFF) and a trailer byte (0xFE), with 320 intermediate bytes encoding high or low voltage levels (3.3 V or 0 V) that control the PIN diodes (SMP 1320-079LF) of each meta-atom. These voltage control signals generated by the FPGA are used to independently control the meta-atoms via a serial-to-parallel shift register (M74HC595). A transmitting horn antenna (HD-58SGAH10) is connected to Port #1 of a vector network analyzer (Ceyear 3672 C 10 MHz~43.5 GHz) via coaxial cable. The transmitting antenna radiates a plane wave with a power of 20 dBm over a frequency range of 4.5–6.5 GHz. Four receiving horn antennas (HD-58SGAH10), positioned at distinct locations, detect the far-field intensities. These antennas are connected via coaxial cables to an RF switch (HMC 321), which routes the signals to a low-noise amplifier (XQY LNA-0.02/10-G22-12V-SE). The amplified signals are transmitted Port #2 of the vector network analyzer. The measured S21 parameters are then sent to the computer over Ethernet.

### Observation and action encoding

#### Representation network

In the optimization of distributed metasurfaces, the input of the representation network includes the latest encoded recognition image of the third-person observations, along with the phase distributions of the distributed metasurfaces from the eight most recent timesteps. The recognition image, a concatenation of the original image and mask, both as RGB images of resolution 256 × 256 and rescaled to the [0,1] range, undergoes no normalization, whitening or other preprocessing. The recognition image is encoded by a CNN into four 8 × 8 planes, while the phase distributions of the distributed metasurfaces are encoded in five 8 × 8 planes, with each plane representing an individual metasurface. Note that the recognition network (Mask-RCNN) is adept at classifying and recognizing 2D images, the modeling of more intricate environments demands the use of advanced models, such as PointNet, to capture the nuances of 3D space with precision. In this context, the 3D point cloud must first be encoded into 8 × 8 planes with multiple channels, then fed into the representation network for further processing.

#### Dynamics network

The input of the dynamics network is the internal state output by the representation network or previous application of the dynamics network, concatenated with a representation of the action for transition. Actions are spatially encoded in planes with the same resolution as the internal state, consistent with the metasurface size of 8 × 8. Two planes are used to encode the action. The first plane indicates whether to place (all-one) or retract (all-zero) a piece, while the second one-hot plane encodes the position of the piece.

By mapping the board game to metasurface optimization, placing a piece means changing the phase state of meta-atom from 0 to π, while retracting a piece means changing its phase from π to 0.

### Monte Carlo tree search

We employ MCTS with upper confidence bounds (UCB) to explore hypothetical future trajectories of phase shifts, which is a planning algorithm tailored for decision-making in uncertain environments. Each node in the search tree corresponds to an internal state *s*. Each action *a* from *s* is linked by an edge (*s*, *a*) that stores a set of statistics {*N*(*s*, *a*), *P*(*s*, *a*), *Q*(*s*, *a*), *R*(*s*, *a*), *S*(*s*, *a*)}, signifying visit counts *N*, policy *P*, mean value *Q*, reward *R* and state transition *S*, respectively.

The search unfolds through three stages: selection, expansion, and backup, each iterated across numerous simulations for comprehensive exploration.

#### Selection

Each simulation starts from the internal root state ${s}_{t}^{0}$, and finishes upon reaching any leaf node ${s}_{t}^{l}$. At each hypothetical step *k* = 1, *…*, *l* of the simulation, an action ${a}_{t}^{k}$ is chosen based on the stored statistics for the internal state ${s}_{t}^{k-1}$, by maximizing over a probabilistic upper confidence tree (PUCT) bound^42^

1 $${{a}}_{{t}}^{{k}}=\text{arg}\mathop{\max }\limits_{a}\{{Q}({s}{,}\,{a})+{P}({s}{,}\,{a})\frac{\sqrt{{\sum }_{{b}}{N}({s}{,}\,{b})}}{1+{N}({s}{,}\,{a})}[{{c}}_{1}+\,\log \left(\frac{{\sum }_{{b}}{N}({s}{,}\,{b})+{{c}}_{2}+1}{{{c}}_{2}}\right)]\}$$

where *a* and *b* represent possible actions. The constants *c*_*1*_ and *c*_*2*_ are used to regulate the impact of the policy *P*(*s, a*) relative to the value *Q*(*s, a*) as nodes are visited more often. In our experiment setup, *c*_*1*_ = 1.25 and *c*_*2*_ = 19,652.

For *k* < *l*, the next internal state ${s}_{t}^{k}$ and reward ${r}_{t}^{k}$ are retrieved from the reward and state transition table ${r}_{t}^{k}$ = *R*(${s}_{t}^{k-1}$, ${a}_{t}^{k}$), ${s}_{t}^{k}$ = *S*(${s}_{t}^{k-1}$, ${a}_{t}^{k}$).

#### Expansion

At the final step *l* of the simulation, a new leaf node is added to the search tree. The reward ${r}_{t}^{l}$ and internal state ${s}_{t}^{l}$ of the new node are calculated using the dynamics network *g*_*ϑ*_, i.e., ${r}_{t}^{l}$,${s}_{t}^{l}$ = *g*_*ϑ*_(${s}_{t}^{l-1}$,${a}_{t}^{l}$), and stored in the corresponding tables, *R*(${s}_{t}^{l-1}$,${a}_{t}^{l}$) = ${r}_{t}^{l}$,*S*(${s}_{t}^{l-1}$,${a}_{t}^{l}$) = ${s}_{t}^{l}$. The policy ${p}_{t}^{l}$ and value ${v}_{t}^{l}$ are calculated using the prediction network *f*_*ϑ*_, i.e., ${p}_{t}^{l},{v}_{t}^{l}={f}_{\theta }({s}_{t}^{l})$. Each edge (${s}_{t}^{l-1}$,${a}_{t}^{l}$) from the newly expanded node is initialized to {*N*(${s}_{t}^{l-1}$,${a}_{t}^{l}$) = 0,*Q*(${s}_{t}^{l-1}$,${a}_{t}^{l}$) = 0, *P*(${s}_{t}^{l-1}$,${a}_{t}^{l}$) = ${p}_{t}^{l},R({s}_{t}^{l-1},{a}_{t}^{l})={r}_{t}^{l},S({s}_{t}^{l-1},{a}_{t}^{l})={s}_{t}^{l}$}. Through looking up statistics in tables, the search algorithm makes at most one call to the dynamics and prediction networks respectively per simulation, significantly reducing computational costs.

#### Backup

At the end of each simulation, the statistics along the trajectory are updated. For *k* = *l*, …, 0, a cumulative discounted reward is calculated

2 $${{G}}_{{t}}^{{k}}=\mathop{\sum }\limits_{{\tau }=0}^{{l}-{k}-1}{{r}}_{{t}}^{{k}+1+{\tau }}+{{v}}_{{t}}^{{l}}$$

In the metasurface optimization, intermediate rewards are not present; the reward is non-zero only at the final step. For *k* = *l*, …, 1, we update the statistics for each edge (${s}_{t}^{k-1}$, ${a}_{t}^{k}$) along the simulation path as follows

3.1 $${Q}({{s}}_{{t}}^{{k}-1},\,{{a}}_{{t}}^{{k}}):=\frac{{N}({{s}}_{{t}}^{{k}-1},\,{{a}}_{{t}}^{{k}})\times {Q}({{s}}_{{t}}^{{k}-1},\,{{a}}_{{t}}^{{k}})+{{G}}_{{t}}^{{k}}}{{N}({{s}}_{{t}}^{{k}-1},\,{{a}}_{{t}}^{{k}})+1}$$

3.2 $${N}({{s}}_{{t}}^{{k}-1},\,{{a}}_{{t}}^{{k}}):={N}({{s}}_{{t}}^{{k}-1},\,{{a}}_{{t}}^{{k}})+1$$

In Eq. (3), we used a variant of the PUCT rule^1^ to amalgamate value estimates with policy probabilities, hence the value is assumed to fall within the [0,1] interval. However, this assumption does not hold true in actual experiments. To restrict the value within the [0,1] range, when a node is reached during the selection stage, the model computes normalized Q-values by employing minimum-maximum values through the following equation

4 $$\bar{Q}({s}_{t}^{k-1},\,{a}_{t}^{k})=\frac{Q({s}_{t}^{k-1},\,{a}_{t}^{k})-\mathop{\min }\limits_{s,a\in Tree}Q(s,\,a)}{\mathop{\max }\limits_{s,a\in Tree}Q(s,\,a)-\mathop{\min }\limits_{s,a\in Tree}Q(s,\,a)}$$

### Data generation

To generate training data, the latest checkpoint of the model (updated every 1,000 training steps) engages in self-play via MCTS, conducting 200 simulations per move to pick an action. During the generation of experience in the metasurface-board games, actions are sampled based on the visit count distribution throughout the duration of each game. Moreover, the visit count distribution is parameterized by a temperature parameter *T*

5 $${\rm{\pi }}({a}{|}{s})=\frac{{N}{({s}{,}{a})}^{1/{T}}}{{\sum }_{{b}}{\rm{N}}({s}{,}\,{b}{)}^{1/{T}}}$$

where *T* is decayed as a function of training steps. Specifically, *T* is set as 1.0 for the initial 150,000 training steps, reduced to 0.5 for the subsequent 150,000 training steps, and then stabilized at 0.25 for the remaining 200,000 training steps. This method ensures that the action selection becomes greedier as training progresses.

Metasurface-board games are sent to the training job as soon as they finish, and the training job keeps an in-memory replay buffer of the latest 125,000 received games.

## Supplementary information

Supplementary file

Supplementary Movie S2

Supplementary Movie S1

## Data Availability

The data that support the findings of this study are available from the authors on reasonable request.
