# Supplementary material for: MetaSeeker: sketching an open invisible space with self-play reinforcement learning
Source: Light Sci Appl. 2025 Jun 4;14:211. doi: 10.1038/s41377-025-01876-0 (PMC12137603; doi:10.1038/s41377-025-01876-0)
Supplement: Supplementary file 1 — Supplementary file [file 41377_2025_1876_MOESM1_ESM.docx]

Supplementary Information for

**MetaSeeker: sketching an open invisible space with self-play reinforcement learning**

Bei Wu^1,2,3^, Chao Qian^1,2,3*^, Zhedong Wang^4^, Pujing Lin^1,2,3^, Erping Li^1,2,3^, and Hongsheng Chen^1,2,3*^

*^1^ZJU-UIUC Institute, Interdisciplinary Center for Quantum Information, State Key Laboratory of Extreme Photonics and Instrumentation, Zhejiang University, Hangzhou 310027, China.*

*^2^ ZJU-Hangzhou Global Science and Technology Innovation Center, Zhejiang Key Laboratory of Intelligent Electromagnetic Control and Advanced Electronic Integration, Hangzhou 310027, China.*

*^3^Jinhua Institute of Zhejiang University, Zhejiang University, Jinhua 321099, China.*

*^4^Zhejiang Province Key Laboratory of Intelligent Vehicle Electronics, Hangzhou Dianzi University, Hangzhou 310018, China.*

*^*^Corresponding authors: chaoq@intl.zju.edu.cn* *(C. Qian);* [*hansomchen@zju.edu.cn*](mailto:hansomchen@zju.edu.cn) *(H. Chen)*

**The PDF file includes:**

Supplementary Note 1: Intelligent EM detector for incident information analysis

Supplementary Note 2: MetaSeeker’s training for metasurface optimization

Supplementary Note 3: Post-hoc interpretability with SHAP

Supplementary Note 4: Vehicular cluster’s system architecture

Supplementary Note 5: MetaSeeker for trajectory planning

**Supplementary Note 1: Intelligent EM detector for incident information analysis**

To analyze unknown incident information, we consider an intelligent EM detector^36^, which is physically made of a hexadecagonal eight-port antenna array. Specifically, the frequency component can be retrieved via the Fourier transform of the time-varying signal. For the polarization and incident angle components, we deployed a generalized regression neural network (GRNN) for inverse reasoning. The predicted information can then serve as input for MetaSeeker to integrate the incident wave knowledge.

**Supplementary Note 2: MetaSeeker’s training for metasurface optimization**

During training, the model is unrolled for *K* hypothetical steps (set as 5 steps) and aligned to sequences sampled from the trajectories generated by the MCTS actors. Sequences are selected by sampling a state from any game in the reply buffer, then unrolling for *K* steps from the state. The model is trained end to end, with three objectives of accurately predicting the policy, value, and reward over k = 0, ..., K steps. The first objective is to minimize the error between the actions predicted by the policy $\text{p}_{\text{t}}^{\text{k}}$ and those by the search policy *π_t+k_*. The second objective is to minimize the error between the predicted immediate reward $\text{r}_{\text{t}}^{\text{k}}$ and the observed reward *u_t+k_*. In the invisibility task without intermediate rewards, the reward exists only at the final step *t* + *N*, with the value of *u_t+N_* = c - $\sum_{\text{i}\text{=0}}^{\text{3}} {\text{(}\text{E}_{\text{i}} \text{- }\hat{\text{E}_{\text{i}}}\text{)}}^{\text{2}}$, where c = 800 is a constant to ensure positive rewards, *E_i_* and $\hat{\text{E}_{\text{i}}}$ are the target and detected far-field intensities at different detection angles, respectively. The game terminates whenever the agent achieves near-perfect invisibility or when the move steps reach a maximum number (set as 320). Unlike MuZero, the agent is allowed to retract pieces in the metasurface-boards, improving its adaptability to dynamic scenarios. The third objective is to minimize the error between the predicted value $\text{v}_{\text{t}}^{\text{k}}$ and the target value *z_t+k_*. For the target value, we bootstrap directly to the end of the metasurface-board game, e.g., *z_t+k_* = $\sum_{\text{τ}\text{=0}}^{\text{N-k-1}} \text{u}_{\text{t+k}\text{+τ}\text{+1}}$, equivalent to predicting the final invisibility performance at each step.

For reward and value prediction, we follow ref. ^2^ in scaling targets using an invertible transform *h*(*x*) = sign(*x*)($\sqrt{\left| \text{x} \right|\text{ + 1}}$ - 1) + *εx*, where *ε* = 0.001 in our experiments. Subsequently, we apply a transformation *ϕ*(*x*) to the scalar reward and value targets to obtain equivalent categorical representations, and use a discrete support set of size 61, with one support for every integer between -30 and 30. Under the transformation *ϕ*(*x*), each scalar *x* is expressed as a linear combination of its adjacent integers *x_low_* and *x_high_*, i.e., *x* = *x_low_* × *p_low_* + *x_high_* × *p_high_*. For instance, a scalar 3.7 would be expressed as a weight of 0.3 on the support for 3 and a weight of 0.7 on the support for 4. To match the target dimension, the reward and value predicted by the network are modelled using a softmax output of size 61. During interference, the actual reward and value are derived by first computing their expected value under their respective softmax distribution and subsequently by inverting the scaling transformation.

Finally, L2 regularization term is added to prevent overfitting of the model, scaled by a constant c, leading to the overall loss

$\text{l}_{\text{t}}\text{(}\text{θ}\text{) = -}\sum_{\text{k}\text{=0}}^{\text{K}} \phi\text{(}\text{z}_{\text{t+k}}\text{)log }\text{v}_{\text{t}}^{\text{k}}\text{-}\sum_{\text{k}\text{=0}}^{\text{K}} \phi\text{(}\text{u}_{\text{t+k}}\text{)log }\text{r}_{\text{t}}^{\text{k}}\text{-}\sum_{\text{k}\text{=0}}^{\text{K}} \text{π}_{\text{t+k}}\text{log }\text{p}_{\text{t}}^{\text{k}}\text{ + c}\left\| \text{θ} \right\|^{\text{2}}$ (6)

**Supplementary Note 3: Post-hoc interpretability with SHAP**

We employ SHAP (Shapley Additive exPlanations) to enhance the transparency of MetaSeeker’s decision-making process. SHAP values, rooted in cooperative game theory, quantify the marginal contributions of each input feature to the model’s prediction. For a model *f*, the SHAP value $\text{ϕ}_{\text{i}}\text{(}\text{f}\text{)}$ is computed as:

$\text{ϕ}_{\text{i}}\text{(}\text{f}\text{) = }\sum_{\text{S}\text{⊆}\text{D}\text{\textbackslash\{}\text{i}\text{\}}} \frac{\text{|}\text{S}\text{|!}\text{ }\text{(|}\text{D}\text{|}\text{ }\text{-}\text{ }\text{|}\text{S}\text{|}\text{ }\text{-}\text{ }\text{1)}\text{!}}{\text{|}\text{D}\text{|!}}\text{(}\text{f}\text{ }\text{(}\text{S}\text{∪}\text{\{}\text{i}\text{\})}\text{ }\text{-}\text{ }\text{f}\text{ }\text{(}\text{S}\text{))}$ (7)

where *D* is the full feature set, *S* is a subset excluding feature *i*. The additive feature attribution framework decomposes the prediction *f*(*x*) into:

$\text{f}\text{(}\text{x}\text{) = }\text{ϕ}_{\text{0}}\text{ +}\sum_{\text{i }\text{=1}}^{\text{ M}} \text{ϕ}_{\text{i}}$ (8)

where $\text{ϕ}_{\text{0}}$ is the baseline prediction, and $\text{ϕ}_{\text{i}}$ reflects the contribution of feature *i*. In our task, the input features are pixel blocks of third-person observations, and $\text{ϕ}_{\text{i}}$ delineates the pixel-level impact on far-field intensities. Visualizing the SHAP values yields the results shown in Fig. 4d of the manuscript.

From the equations above, it is evident that SHAP, as a post-hoc explanatory method, does not participate in model training or architecture design. Instead, it calculates the positive or negative contributions of each input feature after the model f has been trained and the predictions have been made. This makes SHAP independent of the model’s architecture, allowing it to be applied to any black-box model, including linear regression, decision trees, random forests, and deep neural networks.

That SHAP’s computational time is influenced by the complexity and scale of the model (e.g., 38 s per inference for MetaSeeker). However, this computational overhead is distinct from the time required for metasurface optimization, which is significantly faster at just 89ms per iteration. This distinction highlights that the additional time invested in SHAP-based interpretability does not compromise the efficiency of the core optimization process.

Given the multi-modal nature of our inputs, which include metasurface phase patterns and third-person observations, the explanation process is inherently complex. In this context, post-hoc interpretability methods like SHAP provide a practical and effective solution, enabling us to strike a balance between achieving high-performance invisibility and maintaining model interpretability. This trade-off ensures that while we incur additional computational cost for interpretability, the overall system remains efficient and effective in its primary task of metasurface optimization.

**Supplementary Note 4: Vehicular cluster’s system architecture**

Each vehicle in the cluster is equipped with full perception, localization, planning, and control functionalities. The trajectory-broadcasting network serves as the sole connection between individuals, resulting in a low coupling degree for the cluster system. Embracing the single-to-cluster paradigm, a decentralized scheme is naturally established, as illustrated in Fig. 3c, granting each vehicle possesses full autonomy to maximize navigation quality. For perception and localization of each vehicle, the mapping module relies on Gmapping algorithm, and the localization module employs adaptive Monte Carlo Localization (ACML) to compute the position based on laser scans. The trajectory planner that generates high-quality trajectories is the core of vehicular cluster design and therefore is further detailed in the “MetaSeeker for trajectory planning” section in the methods. The trajectory planner continuously chases the latest position target and keeps adjusting the motion policy towards the highest value, and the movement module guides vehicles to follow trajectories devised by the trajectory planner. We conducted three experimental demonstrations: scalability, racing, and tracking.

**Scalability.** As shown in the top image of Fig. 4a, the leader vehicle, distinguished by a pink trajectory, autonomously navigates toward user-defined positions marked by snowflake indicators. It transmits real-time motion control commands to the two slave vehicles through the wireless broadcast network. On the bridge, these commands direct the slave vehicles to specific positions denoted by snowflake markers along their trajectories, with the trajectory planner maintaining its basic settings as depicted in Fig. 3d. When each slave vehicle traverses the right side of the bridge, the control commands mandate maintaining a triangular formation with the slave vehicle. Here, a formation penalty is integrated into the reward function to enforce the triangular arrangement.

**Racing.** The trajectory planner maintains its basic settings while fine-tuning the target positions of the slave vehicle to facilitate effective overtaking, as highlighted with red line in the central image of Fig. 4a. Each vehicle possesses obstacle avoidance capability during autonomous driving. Specifically, in the constrained space on the right side of the bridge (width less than 1.2m), three vehicles, each with a width of 0.21 m, achieve autonomous cluster navigation and overtaking without collisions.

**Tracking**. For dynamic target tracking, a frequency-modulated continuous-wave^3^ (FMCW) lidar is mounted on the vehicle roof, enabling concurrent speed and distance measurements. With specified scan angles, the FMCW lidar identifies the nearest mobile entity in front, disregarding reciprocal vehicles moving on the sides. A tracking penalty is then integrated into the trajectory planner's reward function, guiding three vehicles closely follow a target person while maintaining a specific distance, as depicted in the bottom image of Fig. 4a.

**Supplementary Note 5: MetaSeeker for trajectory planning**

To validate the algorithm’s generality, we extend its application to vehicular trajectory planning, as depicted in Supplementary Fig. 6b. In the vehicular cluster, a leader vehicle orchestrates two slave vehicles. The leader vehicle’s target positions are user-defined, while two slave vehicles’ target positions are dynamically calculated based on leader vehicle’s location and formation requirements. The trajectory planner aims to optimize trajectories for collision-free navigation, which is essentially solving constrained optimization problems. Trajectory planning of vehicular cluster poses a significant challenge because it introduces infinite branching points to the search tree. Thus, we forego the utilization of MCTS for exploring hypothetical future actions, opting instead to directly apply the predicted policy $\text{p}_{\text{t}}^{\text{k}}$ as the subsequent action *a_t+k+1_*, which is a two-dimensional point $\text{p}_{\text{t}}^{\text{k}}$ ∈ ℝ^2^. Note that the trajectory planner decomposes the task of continuous trajectory planning into the planning of multiple discrete positions, exemplified by the red, green, and blue points in Supplementary Fig. 6a, effectively converting continuous-time constrains along the trajectory into a finite sum of conceptualized constraints.

To optimize training efficiency and minimize costs, we construct a high-fidelity digital twin scene using the Gazebo simulation software. In this digital twin, each vehicle’s physical structure, sensors, control system and experimental environment are digitally represented. The digitized vehicle perceives information through laser scans that faithfully reflects real-world situations, constituting the observation *o_t_* of agents. Subsequent steps mirror those in metasurface optimization. Initially, the representation network *h_θ_* takes past observations (*o_1_*, …, *o_t_*) as input and transforms them into the "root" state $\text{s}_{\text{t}}^{\text{0}}$ in its internal world, i.e., $\text{s}_{\text{t}}^{\text{0}}$ = *h_θ_*(*o_1_*, …, *o_t_*). The dynamics network *g_θ_* then iteratively updates the internal state $\text{s}_{\text{t}}^{\text{k}}$ and computes an immediate reward $\text{r}_{\text{t}}^{\text{k}}$ through a recurrent process, taking the previous internal state $\text{s}_{\text{t}}^{\text{k-1}}$ and policy $\text{p}_{\text{t}}^{\text{k-1}}$ as input, i.e., $\text{r}_{\text{t}}^{\text{k}}$, $\text{s}_{\text{t}}^{\text{k}}$ = *g_θ_*($\text{s}_{\text{t}}^{\text{k-1}}$, $\text{p}_{\text{t}}^{\text{k-1}}$). At each step, the prediction network *f_θ_* produces a policy $\text{p}_{\text{t}}^{\text{k}}$ and a value $\text{v}_{\text{t}}^{\text{k}}$, i.e., $\text{p}_{\text{t}}^{\text{k}}$, $\text{v}_{\text{t}}^{\text{k}}$ = *f_θ_*($\text{s}_{\text{t}}^{\text{k}}$).

Likewise, the trajectory planner is trained end to end with three objectives of accurately predicting the policy, value, and reward at every hypothetical step *k*. The first objective is to minimize the error between the predicted immediate reward $\text{r}_{\text{t}}^{\text{k}}$ and observed reward *u_t+k_*. The observed reward *u_t+k_* is a weighted accumulation of target approximation and conceptualized constraints, including time minimization, smoothness maximization, dynamical feasibility, obstacle avoidance, and reciprocal avoidance, as depicted in Fig. 3d. These constraints form the basic settings of the trajectory planner. The second objective is to minimize the error between the predicted value $\text{v}_{\text{t}}^{\text{k}}$ and the target value *z_t+k_*, derived through *n*-step bootstrapping (predefined as 10-step) with discounted rewards, e.g., *z_t+k_* = $\sum_{\text{τ}\text{=0}}^{\text{n}\text{-1}} \text{γ}^{\text{k}}\text{u}_{\text{t+k+τ+1}}\text{ + }\text{γ}^{\text{n}}\text{v}_{\text{t+k+n}}$, where $\text{γ}$ is set as 0.997. This method equips the trajectory planner to factor in future contexts, empowering it to craft decisions with foresight. The third objective is to optimize the policy $\text{p}_{\text{t}}^{\text{k}}$, with its quality manifested through its impact on the subsequent step's value *z_t+k+1_*. The overall loss can be deduced as

$\text{l}_{\text{t}}\text{(}\text{θ}\text{) = -}\text{ }\sum_{\text{k}\text{=0}}^{\text{K}} \phi\text{(}\text{z}_{\text{t+k}}\text{)log }\text{v}_{\text{t}}^{\text{k}}\text{-}\text{ }\sum_{\text{k}\text{=0}}^{\text{K}} \phi\text{(}\text{u}_{\text{t+k}}\text{)log }\text{r}_{\text{t}}^{\text{k}}\text{-}\text{ }\sum_{\text{k}\text{=0}}^{\text{K}} \text{z}_{\text{t+k+1}}\text{+ c}\left\| \text{θ} \right\|^{\text{2}}$ (9)

where c is the scaled factor of the L2 regularization term, and *θ* are parameters of the trajectory planner *μ_θ_*.

After training in the digital twin scene, the trajectory planner is deployed to vehicle entities, guiding them along the planned route. Concurrently, the real-time motion status of each vehicle is mirrored in the digital twin scene, thereby actualizing an integration of the virtual and real worlds.

**Target approximation**. We apply a negative reward based on the distance between the current position and the target position to guide the vehicle towards the destination.

$\text{u}_{\text{t+k}}^{\text{p}}\text{ = - }\left\| \left. \text{p}\text{(}\text{k}\text{) - }\bar{\text{p}}\text{(}\text{k}\text{)} \right\| \right._{\text{2}}^{\text{2}}$ (10)

where ***p***$\text{(}\text{k}\text{)}$/$\bar{\text{p}}\text{(}\text{k}\text{)}$ is the current/target position at step *k*, and ***p***$\text{(}\text{k}\text{)}$ ≈ $\text{p}_{\text{t}}^{\text{k-1}}$

**Time minimization.** We minimize driving time by applying a negative reward as follows

$\text{u}_{\text{t+k}}^{\text{m}}\text{ = - T}$ (11)

where T is the time duration of moving from the current position to the target position at step *k*.

**Smoothness maximization.** To ensure the trajectory smoothness, a penalty is introduced into the reward function, quantified as the squared second derivative.

$\text{u}_{\text{t+k}}^{\text{s}}\text{ }\text{= - }\int_{\text{0}}^{\text{T}} \left\| \left. \ddot{\text{p}}\text{(}\text{t}\text{)} \right\| \right._{\text{2}}^{\text{2}}\text{ }\text{d}\text{t}$ (12)

where ***p***(*t*) ∈ ℝ^2^ represents the trajectory fitted from the planned discrete positions.

**Dynamical feasibility.** To ensure the dynamical feasibility of vehicles, we constrain the amplitude of velocity and acceleration, imposing a penalty if these derivatives surpass predefined physical thresholds.

$\text{u}_{\text{t+k}}^{\text{d,v}}\text{ }\text{= - }{\text{max \{(}{\dot{\text{p}}\text{(}\text{k}\text{)}}^{\text{2}}\text{ - }\text{v}_{\text{m}}^{\text{2}}\text{), 0\}}}^{\text{2}}$ (13.1)

$\text{u}_{\text{t+k}}^{\text{d,a}}\text{ = - }{\text{max \{(}{\ddot{\text{p}}\text{(}\text{k}\text{)}}^{\text{2}}\text{ - }\text{a}_{\text{m}}^{\text{2}}\text{), 0\}}}^{\text{2}}$ (13.2)

where ***v****_m_* and ***a****_m_* represent the maximum magnitudes of velocity and acceleration set as 1.0 m/s and 0.8 m/s^2^, respectively.

**Obstacle and reciprocal avoidance.** The distance *d_0_* between the current position ***p***$\text{(}\text{k}\text{)}$ and obstacles is defined as

$\text{d}_{\text{0}}\text{ = }{\text{(}\text{p}\text{(}\text{k}\text{)}\text{ }\text{-}\text{ }\text{s}\text{)}}^{\text{T}}\text{v}$ (14)

where ***s*** ∈ ℝ^2^ is a point on the obstacle boundary, and ***v*** ∈ ℝ^2^ is a normal vector pointing to the free side. It's worth noting that obstacles in the environment and reciprocal vehicles are both treated as obstacles, with their boundaries identified through laser scans.

Following the definition of *d_0_*, a penalty is imposed if it falls below the expansion radius _0_ (set as 0.3 m), e.g., *d_0_* < _0_. The obstacle avoidance penalty is formulated as

$\text{u}_{\text{t+k}}^{\text{a}}\text{ }\text{= - }{\text{max \{(}\text{}\text{0}\text{ - }\text{d}_{\text{0}}\text{), 0\}}}^{\text{2}}$ (15)

**Formation expectation.** To stay in formation, we employ graph theory to compute the target trajectory for each vehicle. Each vehicle, assigned a vertex, plans its trajectory based on the movements of other vehicles. Subsequently, the frame origin is determined by aligning the formation shape with the current cluster distribution. This formation inference anticipates the formation position in the near future, providing a guiding position ***g***(*k*) for the trajectory planning of each vehicle. The formation penalty is defined as

$\text{u}_{\text{t+k}}^{\text{f}}\text{ }\text{= - }\left\| \left. \text{p}\text{(}\text{k}\text{) - }\text{g}\text{(}\text{k}\text{)} \right\| \right._{2}^{2}$ (16)

According to the provided definition, we can derive the observed reward *u_t+k_* at step *k* as follows

$\text{u}_{\text{t+k}}\text{ = }\sum_{\text{x}} \text{λ}_{\text{x}}\text{u}_{\text{t+k}}^{\text{x}}$ (17)

where $\text{u}_{\text{t+k}}^{\text{x}}$ represents various penalty terms, and $\text{λ}_{\text{x}}$ are relative weights. The subscript *x* = {*p*, *m*, *s*, *d*, *a*, *f*} corresponds to target approximation (*p*), time minimization (*m*), smoothness maximization (*s*), dynamical feasibility (*d*), obstacle and reciprocal avoidance (*a*) and formation expectation (*f*). The specified values for {*λ_p_*, *λ_m_*, *λ_s_*, *λ_d_*, *λ_a_*, *λ_f_*} are {1, 0.1, 0.01, 10, 10, 1}.

**References**

1. Rosin, C. D. Multi-armed bandits with episode context. *Ann. Math. Artif. Intell.* **61**, 203-230 (2011).
2. Pohlen, T. *et al.* Towards consistent performance on atari using expert demonstrations (2018).
3. Liu, J. *et al.* Monolithic piezoelectric control of soliton microcombs. *Nature* **583**, 385-390 (2020).
4. He, K., Zhang, X., Ren, S. & Sun, J. *Identity mappings in deep residual networks*, 630-645 (Springer, 2016).


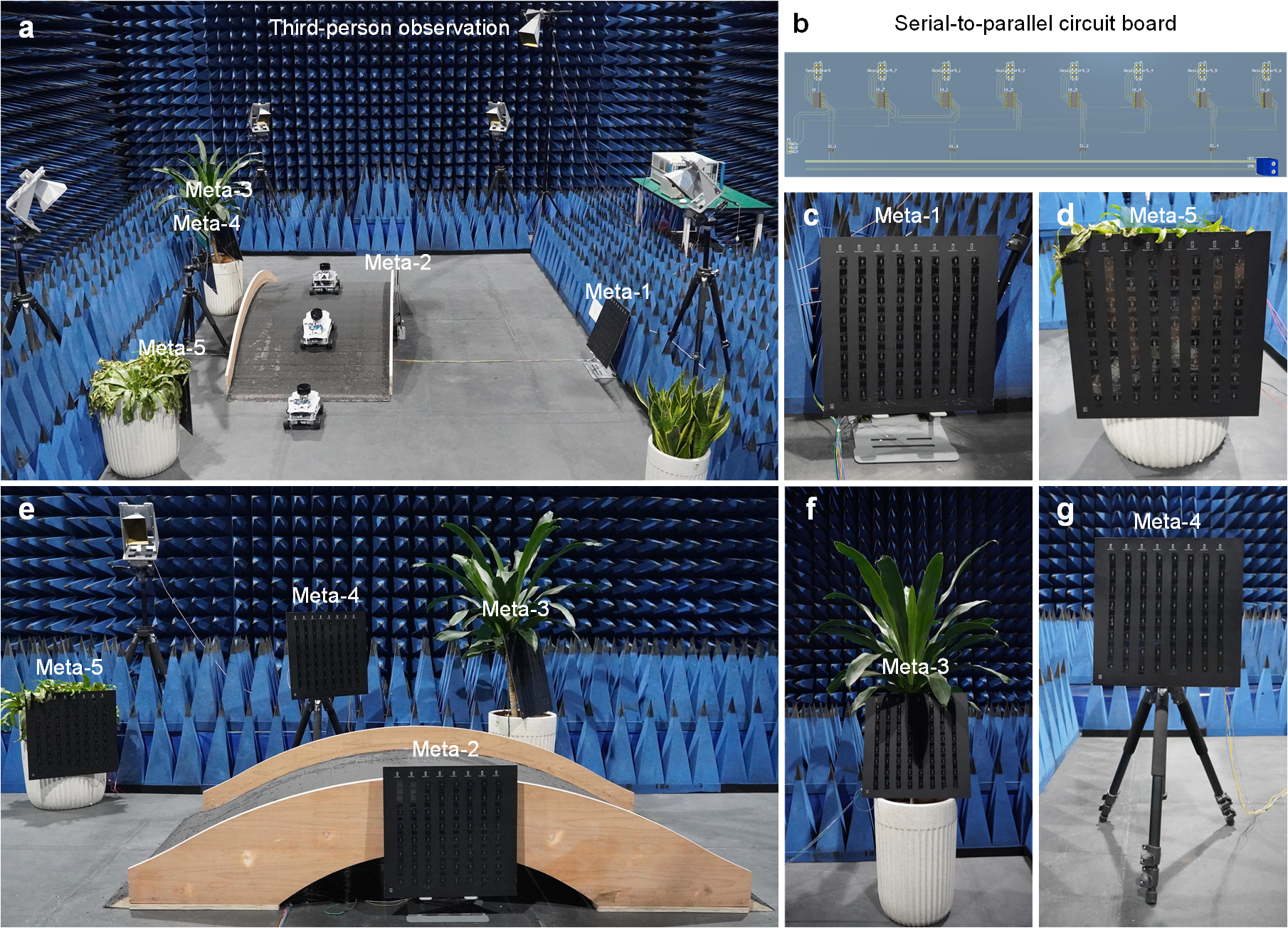


**Supplementary Fig. 1 | Detailed images and controls of the distributed metasurfaces. a**, Third-person observation of the experimental scene, comprising five metasurfaces. **b**, Seriral-to-parallel circuit board connected behind metasurface. Eight 74HC595 chips are welded inside the circuit board, transforming 8-bits sertial intputs into parallel outputs. This configuration enables independent control of all meta-atoms using only three signal lines: serial clock, register clock and serial intput. **c-g**, Detailed images of metasurfaces.


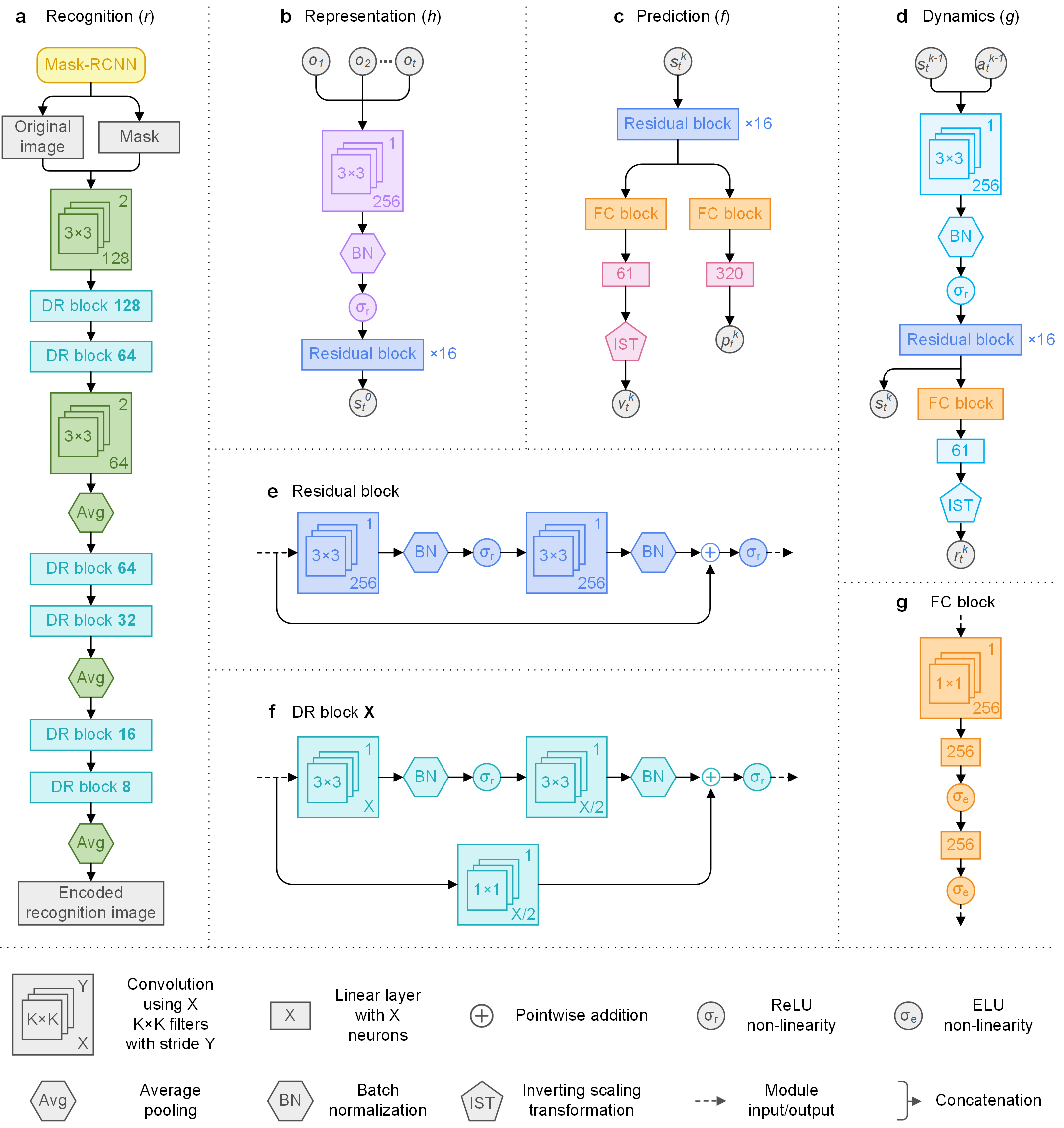


**Supplementary Fig. 2 | Architectures of recognition, representation, dynamics, and prediction networks. a**, The recognition network (*r*) receives the original image of experimental scene and the mask output from Mask-RCNN, both as RGB images of resolution 256 × 256 and rescaled to the [0,1] range. The concatenation of original image and mask is then encoded into four 8 × 8 planes, consistent with the metasurface size. **b**, The representation network (*h*) receives the latest encoded recognition image output from the recognition network, along with the phase distributions of the distributed metasurfaces from the eight most recent timesteps. It transforms the past observations into the "root" internal state of resolution 8 × 8 and 256 planes. **c**, The prediction network (*f*) produces a policy and a value for an internal state. The policy is a one-dimensional tensor of size 320, matching the total number of meta-atoms in the distributed metasurfaces. The predicted value is a one-dimensional tensor of size 61, aligning with the dimension of target after scaling transformation (see "MetaSeeker’s training for metasurface optimization" in methods). **d**, The dynamics network (*g*) receives the previous internal state and a hypothetical next action, then updates the internal state and computes an immediate reward. The input action has a resolution of 8 × 8 and two planes. Similar to value prediction, the reward is also a one-dimensional tensor of size 61. **e**, Residual block^4^. **f**, Dimension reduction (DR) block remains the input’s resolution while halving the number of planes. **g**, Fully connected (FC) block.


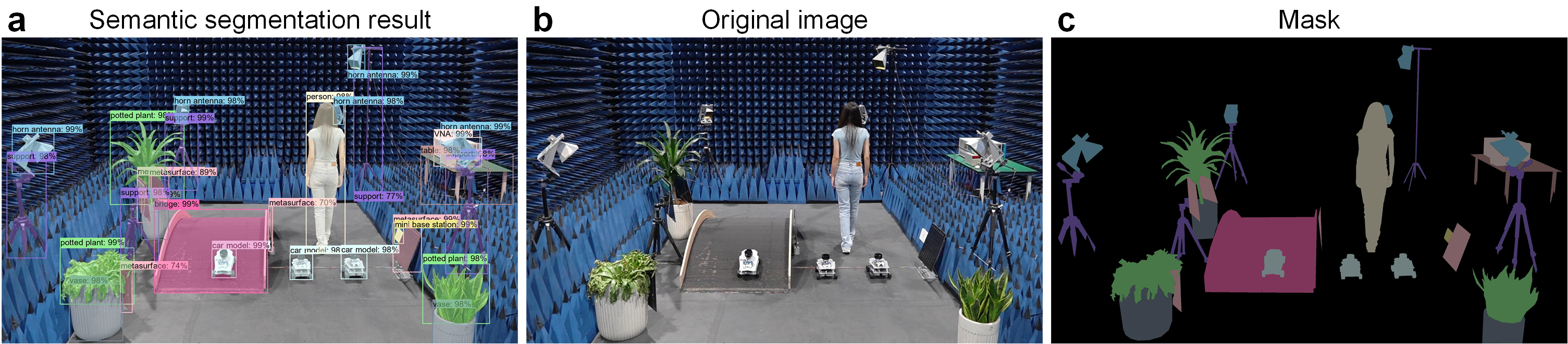


**Supplementary Fig. 3 |** **a**, Semantic segmentation result, **b**, original image and **c**, mask of the third-person observation. The mask indicates the categories, positions and contours of interferences, enriching the agent's comprehension of the experimental environment.


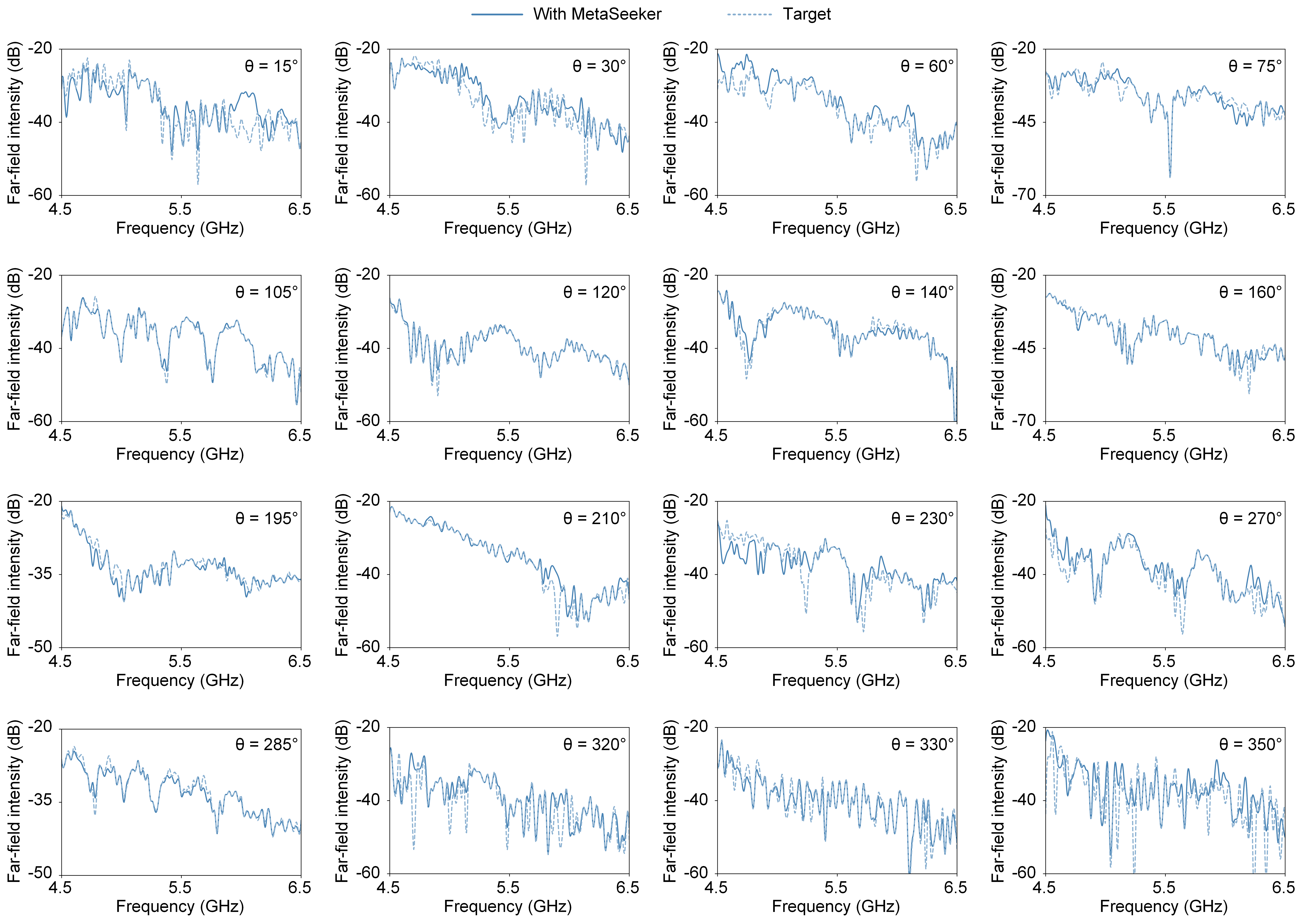


**Supplementary Fig. 4 | More experimental results of broadband invisibility at different detection angles.** The solid line represents the far-field intensities optimized by MetaSeeker, and the dashed line represents the target.


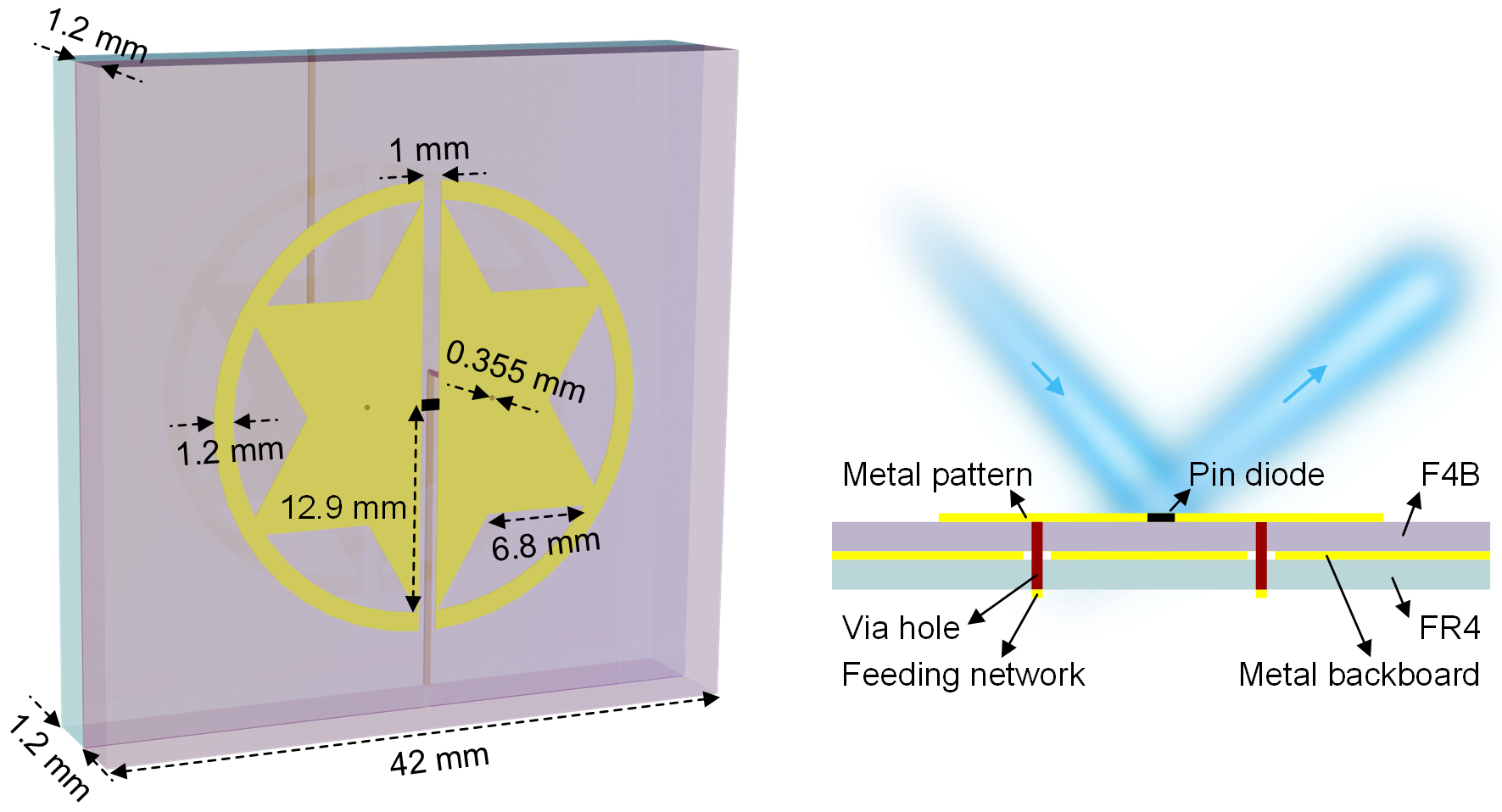


**Supplementary Fig. 5 |** The detailed structure of meta-atom, which comprises two substrate layers: an F4B layer (ε = 2.2) and an FR4 layer (ε = 4.3), separated by a metal backboard for high reflection amplitude. A pin diode (SMP 1320-079LF) is connected between the adjacent metal patterns on the top layer. It is biased by a feeding network printed on the bottom of FR4 substrate, with via holes connecting the metal patterns and the feeding network.


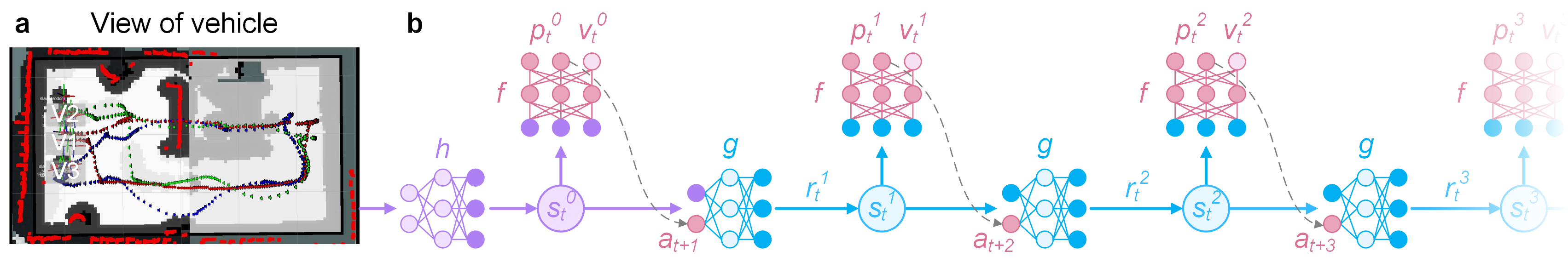


**Supplementary Fig. 6 | Architecture of MetaSeeker for trajectory planning**. **a**, Visualization of the internal world of vehicles. Each vehicle in the cluster perceives its surroundings through laser scans, constituting the observation *o_t_* of agents. **b**, The model of MetaSeeker for trajectory planning, applicable to continuous action domains.
